# Effect of Neferine on DNCB-Induced Atopic Dermatitis in HaCaT Cells and BALB/c Mice

**DOI:** 10.3390/ijms22158237

**Published:** 2021-07-30

**Authors:** Chung-Chi Yang, Yen-Ling Hung, Wen-Chin Ko, Yi-Ju Tsai, Jia-Feng Chang, Cher-Wei Liang, Der-Chen Chang, Chi-Feng Hung

**Affiliations:** 1 Division of Cardiovascular Medicine, Taoyuan Armed Forces General Hospital, Taoyuan City 32551, Taiwan; t220979@gmail.com; 2 Graduate Institute of Biomedical and Pharmaceutical Science, Fu Jen Catholic University, New Taipei City 24205, Taiwan; irishung0919@gmail.com (Y.-L.H.); 085027@mail.fju.edu.tw (Y.-J.T.); 3 School of Medicine, Fu Jen Catholic University, New Taipei City 24205, Taiwan; 054317@gmail.com (W.-C.K.); cjf6699@gmail.com (C.-W.L.); 4 Division of Cardiac Electrophysiology, Department of Cardiovascular Center, Cathay General Hospital, Taipei 10630, Taiwan; 5 Department of Internal Medicine, Division of Nephrology, En Chu Kong Hospital, New Taipei City 23702, Taiwan; wck@cgh.org.tw; 6 Department of Mathematics and Statistics and Department of Computer Science, Georgetown University, Washington, DC 20057, USA; 409466019@mail.fju.edu.tw; 7 PhD Program in Pharmaceutical Biotechnology, Fu Jen Catholic University, New Taipei City 24205, Taiwan; 8 Department of Fragrance and Cosmetic Science, Kaohsiung Medical University, Kaohsiung 80708, Taiwan

**Keywords:** natural product, neferine, keratinocytes, atopic dermatitis, MAPK, NF-κB

## Abstract

Atopic dermatitis (AD) is a chronic and persistent inflammatory skin disease characterized by eczematous lesions and itching, and it has become a serious health problem. However, the common clinical treatments provide limited relief and are accompanied by adverse effects. Therefore, there is a need to develop novel and effective therapies to treat AD. Neferine is a small molecule compound isolated from the green embryo of the mature seeds of lotus (*Nelumbo nucifera*). It has a bisbenzylisoquinoline alkaloid structure. Relevant studies have shown that neferine has many pharmacological and biological activities, including anti-inflammatory, anti-thrombotic, and anti-diabetic activities. However, there are very few studies on neferine in the skin, especially the related effects on inflammatory skin diseases. In this study, we proved that it has the potential to be used in the treatment of atopic dermatitis. Through in vitro studies, we found that neferine inhibited the expression of cytokines and chemokines in TNF-α/IFN-γ-stimulated human keratinocyte (HaCaT) cells, and it reduced the phosphorylation of MAPK and the NF-κB signaling pathway. Through in vivo experiments, we used 2,4-dinitrochlorobenzene (DNCB) to induce atopic dermatitis-like skin inflammation in a mouse model. Our results show that neferine significantly decreased the skin barrier damage, scratching responses, and epidermal hyperplasia induced by DNCB. It significantly decreased transepidermal water loss (TEWL), erythema, blood flow, and ear thickness and increased surface skin hydration. Moreover, it also inhibited the expression of cytokines and the activation of signaling pathways. These results indicate that neferine has good potential as an alternative medicine for the treatment of atopic dermatitis or other skin-related inflammatory diseases.

## 1. Introduction

*Nelumbinis plumula* is the green embryo of the mature seeds of *Nelumbo nucifera* Gaertn. The earliest documented record of *Nelumbinis plumula* is in the “Food Nature Materia Medica” from the late Tang Dynasty [1]. The “Compendium of Materia Medica” of the Ming Dynasty records *Nelumbinis plumula* as “bitter, cold, non-toxic”. Over the years, whether as a medicine or as a health tea application, it has been proven that *Nelumbinis plumula* does have unique effects. According to Pharmacopoeia, dried *Nelumbinis plumula* contains no less than 0.2% alkaloids. The current alkaloid content is used as a standard for the quality evaluation of *Nelumbinis plumula* [1]. According to previous research reports, the main alkaloids in *Nelumbinis plumula* are liensinine, isoliensinine, and neferine, among which the content of neferine is the highest. Recent studies have demonstrated that neferine effectively prevents UV-induced skin photoaging and photodamage [2]. The results suggest that neferine has strong antioxidative and photoprotective properties and, thus, may be a potential agent for the prevention and treatment of UVA-mediated skin photoaging [3]. Neferine increased the cell viability by enhancing intracellular antioxidants and protected keratinocytes against UVB-mediated increases in ROS and lipid peroxidation [4]. The above results show that neferine has the potential to treat and prevent diseases in the skin. The prevalence of allergic and inflammatory skin diseases in developing countries is very high. Among them, atopic dermatitis and contact allergic dermatitis are still treated and controlled by steroids and calcinurin receptor inhibitors as the main drug treatments. However, these diseases remain incurable and patients often relapse [5]. Although biological agents have recently been developed whose therapeutic effects are very good, their high price still limits their accessibility [6]. Therefore, there is an urgent need to develop compounds that are reasonably priced and effective. The pathological or symptomatic production in both atopic dermatitis and contact dermatitis is closely related to keratinocytes, immune cells, and nerves. Among them, keratinocytes, which release many different cytokines and chemokines after being stimulated, play a very important role [7,8]. Therefore, compounds that affect the function of keratinocytes will have therapeutic potential. Many studies have shown that natural ingredients, such as isoflavones, apigenin, quercetin, and chrysin, have very good pharmacological activities in this regard [9,10,11,12,13,14]. Neferine has been found to inhibit the production or release of inflammatory substances, cytokines, and chemokines in other tissues or cells. The inhibitory effect on amiodarone-induced pulmonary fibrosis, probably due to its anti-inflammation, SP-D inhibition, and SP-D restoration properties, increased CD4+CD25+ Tregs, which may modulate a Th1/Th2 imbalance by suppressing the Th2 response [15]. Neferine can attenuate bleomycin-induced pulmonary fibrosis. This effect may be related to its anti-inflammatory and antioxidant, cytokine, and NF-κB inhibitory activities [16]. However, there is no relevant research on neferine against inflammatory skin diseases. In this study, TNF-α and INF-γ were used to stimulate keratinocytes to study its related mechanisms, and mice were stimulated with DNCB to study the effectiveness of neferine. We found that neferine is a compound with great potential for the treatment of allergic and inflammatory skin diseases.

## 2. Results

### 2.1. Cell Viability in Human Keratinocyte (HaCaT) Cells under Different Concentrations of Neferine Treatment

**Figure 1:**
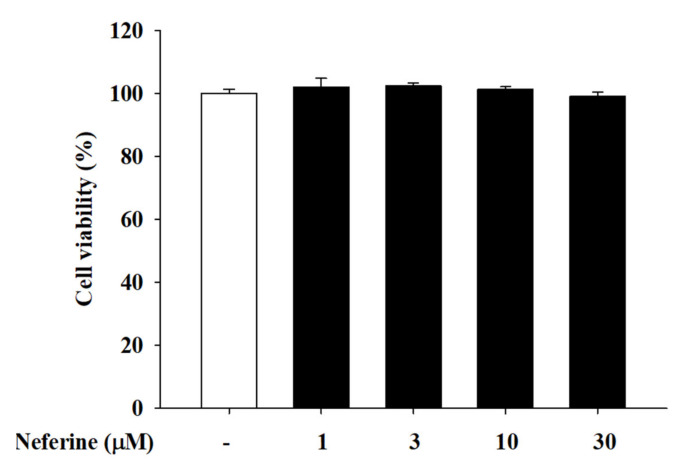
Cell viability of HaCaT cells after pretreatment with different concentrations of neferine (1, 3, 10, and 30 μM). HaCaT cells were treated with different concentrations of neferine at 37 °C for 24 h in 5% CO_2_. Values represent the mean ± SEM from at least three experiments.

To evaluate the effect of neferine on cell viability, we conducted a MTT assay after treating HaCaT cells with 1, 3, 10, and 30 μM of neferine for 24 h. No cytotoxicity was observed at concentrations as high as 30 μM (Figure 1). Therefore, we focused on treatment with 1, 3, and 10 μM of neferine and tested the biological activity and pharmacological mechanism of neferine in the skin.

### 2.2. Neferine Reduced the mRNA Expression of Pro-Inflammatory Cytokines and Chemokines in TNF-α/IFN-γ-Stimulated HaCaT Cells

**Figure 2:**
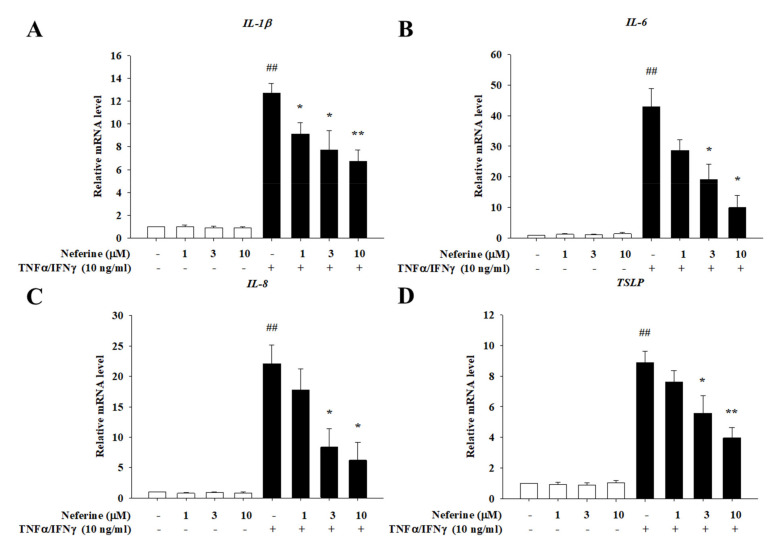
The effect of neferine on the mRNA expression levels of cytokines in TNF-α/IFN-γ-stimulated HaCaT cells. HaCaT cells were pretreated with different concentrations of neferine (1, 3, and 10 μM) for 20 min, and then the cells were treated with TNF-α/IFN-γ (10 ng/mL) for 1 h or 24 h (for TSLP). Total RNA was isolated, and the mRNA expression level of (**A**) IL-1β, (**B**) IL-6, (**C**) IL-8, and (**D**) TSLP was determined using qPCR. Values represent the mean ± SEM from three independent experiments. ^##^ *p* < 0.01 compared with the no-treatment condition; * *p* < 0.05 and ** *p* < 0.01 compared with the TNF-α/IFN-γ treatment condition.

**Figure 3:**
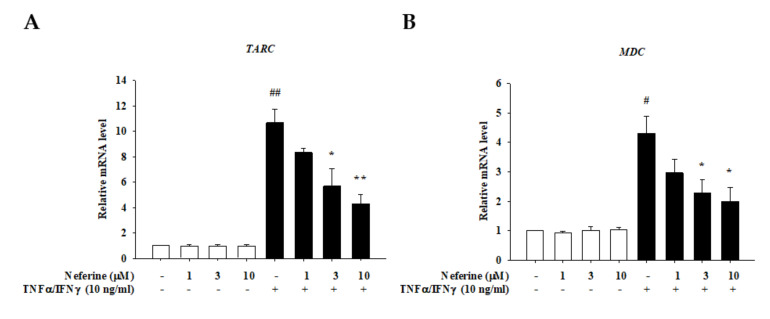

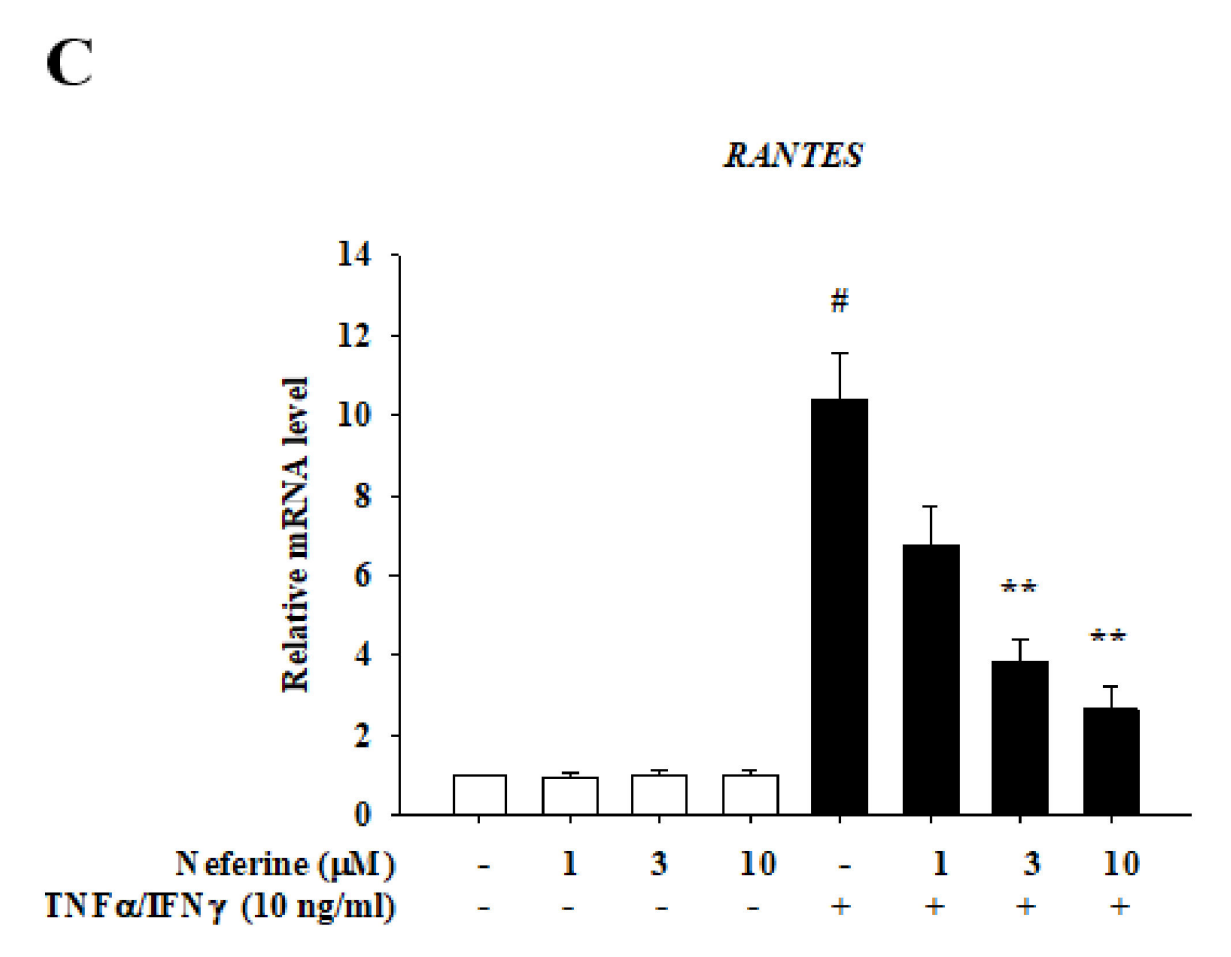
The effect of neferine on the mRNA expression levels of chemokines in TNF-α/IFN-γ-stimulated HaCaT cells. HaCaT cells were pretreated with different concentrations of neferine (1, 3, and 10 μM) for 20 min, and then the cells were treated with TNF-α/IFN-γ (10 ng/mL) for 24 h. The mRNA expression level of (**A**) TARC (CCL17), (**B**) MDC (CCL22), and (**C**) RANTES was determined using qPCR. Values represent the mean ± SEM from three independent experiments. ^#^ *p* < 0.05, ^##^ *p* < 0.01 compared with the no-treatment condition; * *p* < 0.05 and ** *p* < 0.01 compared with the TNF-α/IFN-γ treatment condition.

Stimulation of HaCaT cells by TNF-α/IFN-γ is widely used to find potential candidates for the treatment of atopic dermatitis [17]. The inflammatory factors TNF-α and IFN-γ in the skin of a patient with atopic dermatitis can activate the patient’s keratinocytes to produce an overexpression of inflammatory factors [18]. These cytokines contribute to the infiltration of inflammatory cells into dermal skin [19]. Therefore, we examined the role of neferine in TNF-α/IFN-γ-treated HaCaT cells to investigate whether neferine is a promising agent for the treatment of atopic dermatitis. Previous studies have shown that TNF-α/IFN-γ acts on keratinocytes. After its receptor is activated, it induces the production of IL-1β, IL-6, IL-8, TSLP, TARC, MDC, and RANTES, causing skin-related inflammation. Therefore, in this study, we used neferine to pretreat HaCaT cells for 20 min, then stimulated the cells with TNF-α/IFN-γ for 1 h or 24 h, and finally used RT-qPCR to determine whether neferine affects the mRNA expression of these cytokines and chemokines. The results show that, in the group treated with neferine alone, the mRNA expression of cytokines was not affected and the TNF-α/IFN-γ significantly increased the mRNA expression. In the group treated with neferine, it was observed that the mRNA expression levels of IL-1β, IL-6, IL-8, TSLP, TARC, MDC, and RANTES were all suppressed. Neferine was found to have a significant inhibitory effect at the concentration levels of 3 μM and 10 μM (Figure 2 and Figure 3, respectively).

### 2.3. Neferine Reduced MAPK Activation in TNF-α/IFN-γ-Stimulated HaCaT Cells

**Figure 4:**
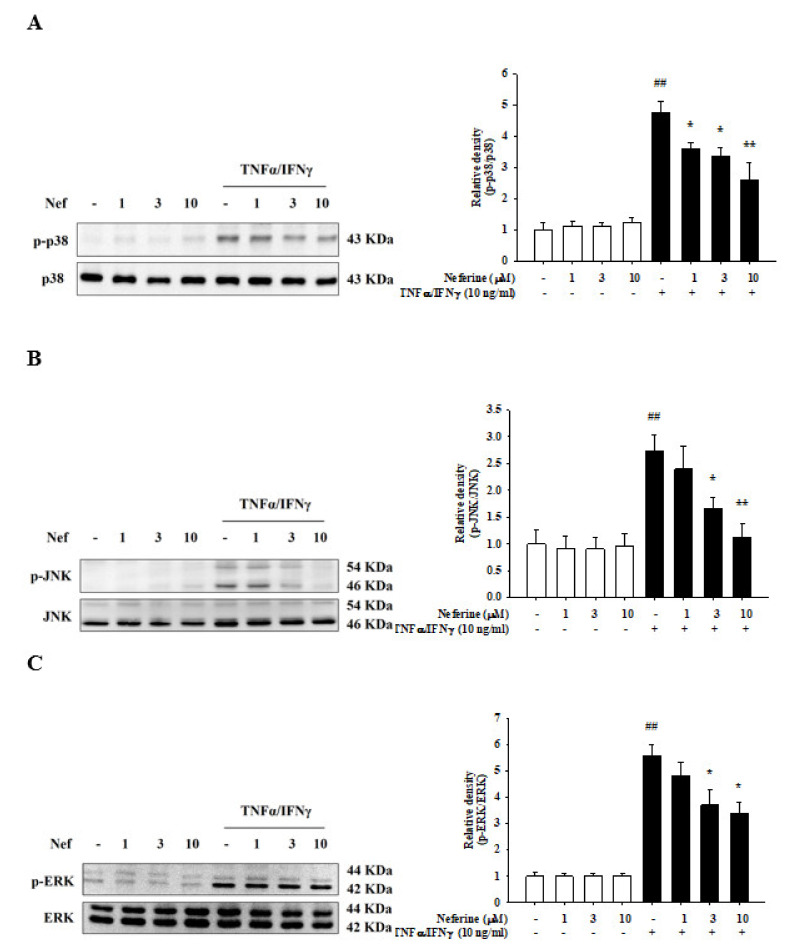
Neferine reduced TNF-α/IFN-γ-induced JNK (**A**), p38 (**B**), and ERK (**C**) activation in human keratinocyte (HaCaT) cells. Human keratinocyte (HaCaT) cells were pretreated with different concentrations of neferine (1, 3, and 10 μM) for 20 min, and then the cells were treated with TNF-α/IFN-γ (10 ng/mL) for 30 min. Western blots were analyzed quantitatively. Values represent the mean ± SEM from three independent experiments. ^##^ *p* < 0.01 compared with the no-treatment condition; * *p* < 0.05 and ** *p* < 0.01 compared with the TNF-α/IFN-γ treatment condition.

The MAPK signaling pathway is closely related to the activation of TNFα/IFNγ, and it regulates various reactions downstream of the entire keratinocyte [20]. In order to study the anti-inflammatory mechanism of neferine, we determined the expression of MAPKs and NF-kB pathways, which are the main inflammatory mediators of atopic dermatitis. Western blot was used to analyze the protein phosphorylation of p38, JNK, and ERK. The experimental results show that treatment of HaCaT cells with different concentrations of neferine alone did not affect the activation of proteins. TNF-α/IFN-γ significantly induced the phosphorylation of p38, JNK, and ERK. In the group of HaCaT cells pretreated with neferine, it was observed that the phosphorylation of the proteins gradually decreased. The band density was analyzed quantitatively, and the concentrations of 3 μM and 10 μM were found to have had a significant inhibitory effect on the protein phosphorylation (Figure 4).

### 2.4. Neferine Reduced IκB and NF-κB Activation in TNF-α/IFN-γ-Stimulated HaCaT Cells

**Figure 5:**
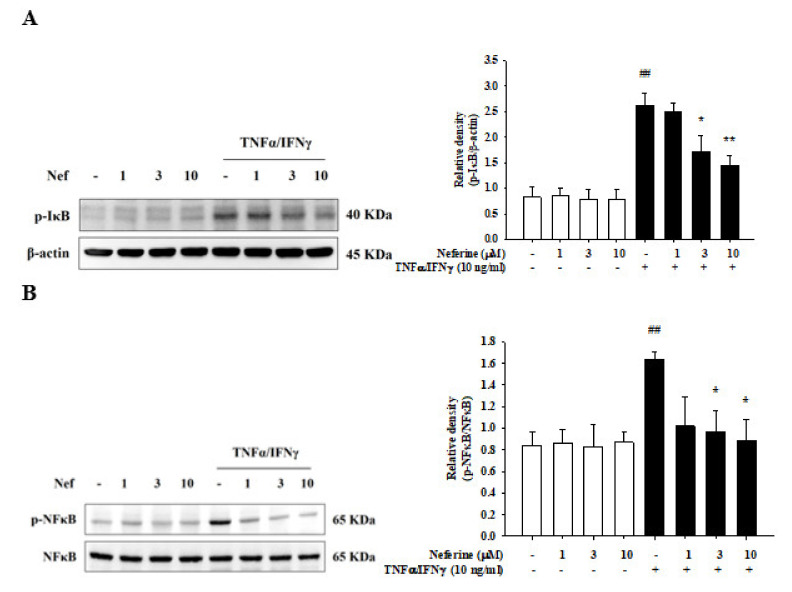
Neferine inhibited TNF-α/IFN-γ-induced IκB (**A**) and NF-κB (**B**) activation in HaCaT cells. Cells were pretreated with different concentrations of neferine (1, 3, and 10 μM) for 20 min and then were treated with TNF-α/IFN-γ (10 ng/mL) for 30 min. Western blots were analyzed quantitatively. Values represent the mean ± SEM from three independent experiments. ^##^ *p* < 0.01 compared with the no-treatment condition; * *p* < 0.05 and ** *p* < 0.01 compared with the TNF-α/IFN-γ treatment condition.

Activation of NF-κB and IκB also leads to the production of cytokines and chemokines and regulates the immune response, cell degradation, cell differentiation, and inflammation [21]. Therefore, we determined whether the protein phosphorylation induced by TNF-α/IFN-γ-stimulated inflammation had decreased after pretreatment with neferine. The results show that pretreatment of HaCaT cells with different concentrations of neferine alone did not affect the phosphorylation of IκBα and NF-κB. Stimulation with TNF-α/IFN-γ produced a significant increase in IκBα and NF-κB phosphorylation. It was observed that the phosphorylation of IκBα and NF-κB was decreased after pretreatment with neferine in TNF-α/IFN-γ-stimulated HaCaT cells (Figure 5).

### 2.5. The Effect of Neferine on the Skin Appearance in DNCB-Induced BALB/c Mice

**Figure 6:**
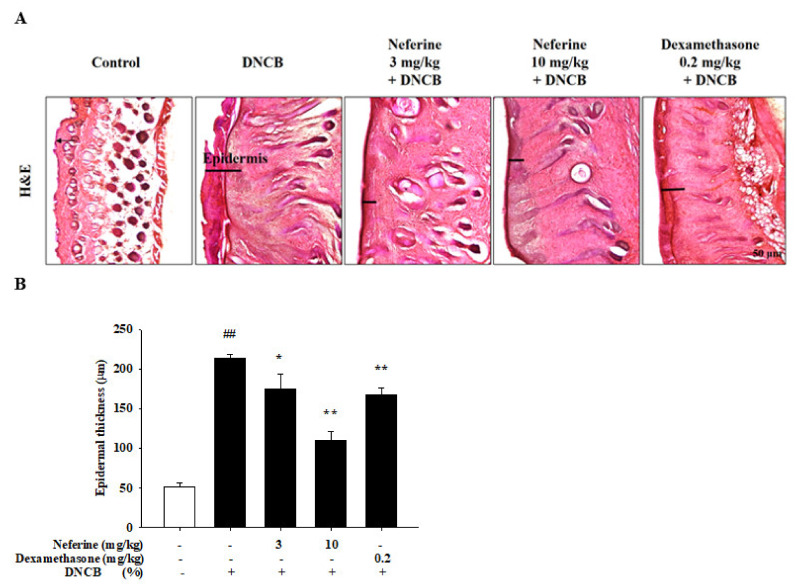
Neferine ameliorated skin inflammation in DNCB-induced BALB/c mice. (**A**) Histopathological variation due to DNCB induction was evaluated using hematoxylin–eosin staining (scale bar, 50 μm). (**B**). Quantitative analysis of epidermal thickness. Values represent the mean ± SEM from at least six independent experiments. ^##^ *p* < 0.01 compared with the no-treatment condition; * *p* < 0.05 and ** *p* < 0.01 compared with the DNCB-induced group.

By repeatedly exposing the dorsal skin area of mice to DNCB, AD-like skin damage was induced. During the experiment, we recorded the changes in the appearance of the skin on the back and ears of the mice (Figure S1). We found that the dorsal skin and ears of the mice in the DNCB-induced group had serious redness, inflammation, and desquamation, and the mice in the neferine treatment group had reduced redness and inflammation. Hematoxylin and eosin staining was used to assess the histopathological changes induced by DNCB. In the DNCB group, the epidermis was abnormally thickened. Compared with DNCB-induced mice, the histological characteristics of the skin in the neferine and dexamethasone treatment groups were improved (Figure 6A,B).

### 2.6. Change in Physiological Parameters of DNCB-Induced BALB/c Mouse Skin after Treatment with Neferine

**Figure 7:**
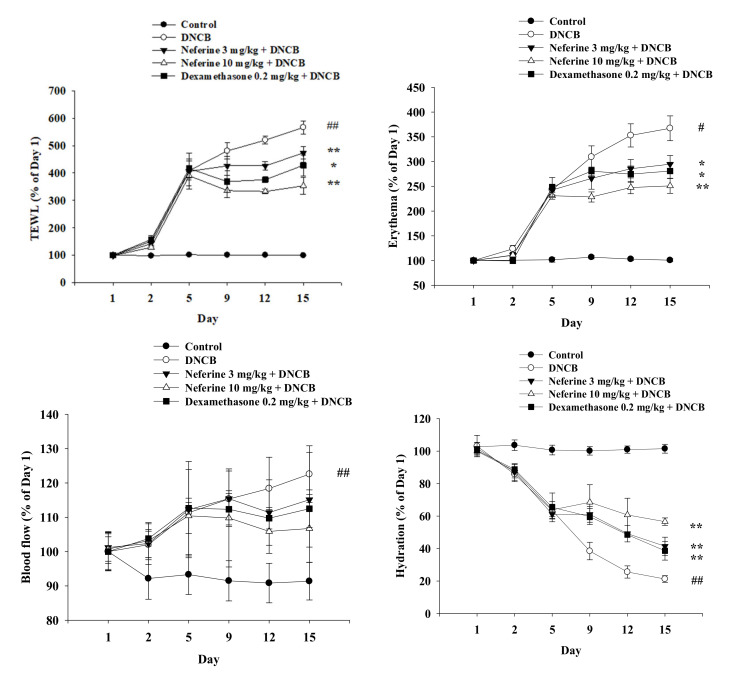

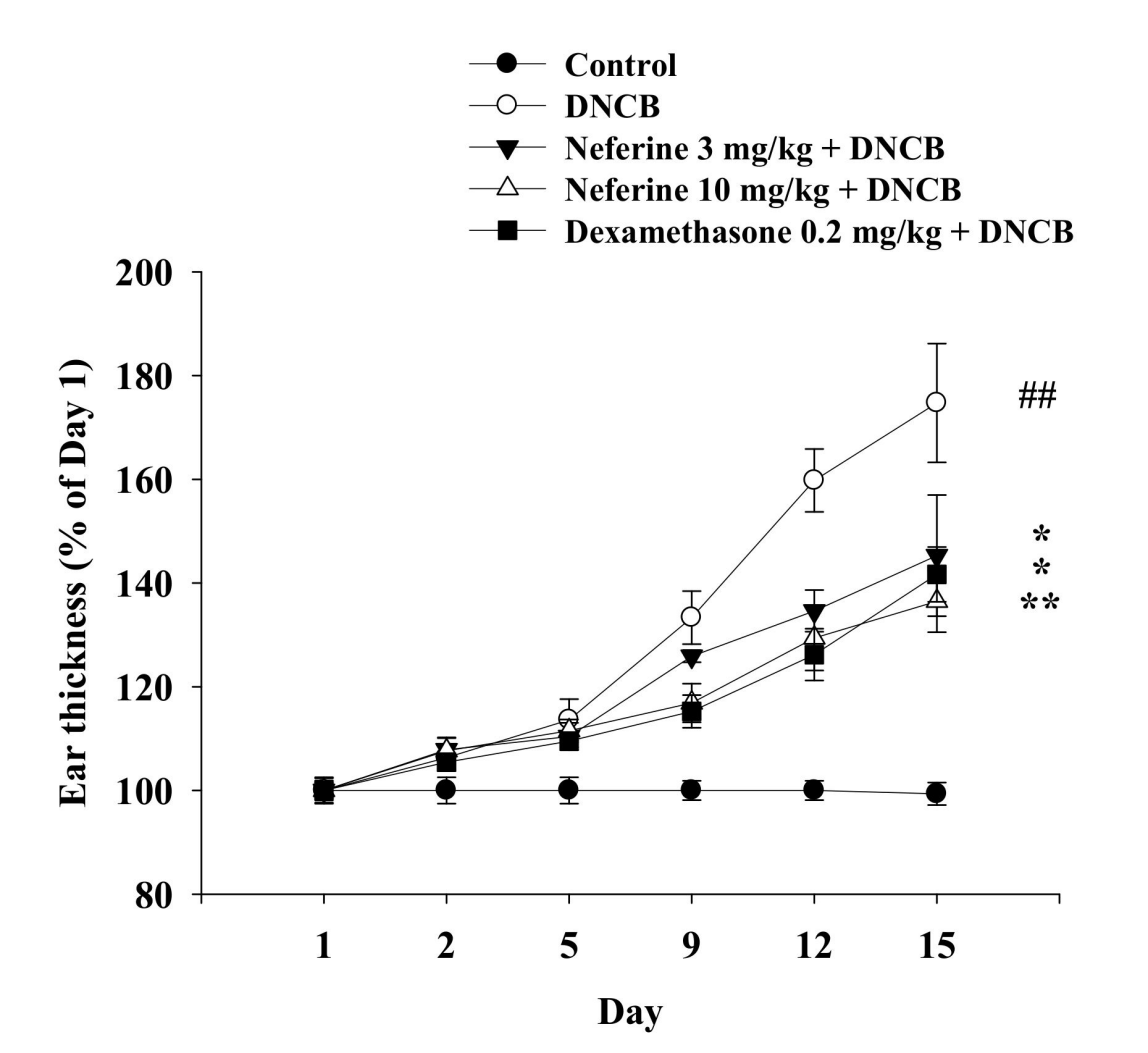
Change in physiological parameters of DNCB-induced BALB/c mouse skin after treatment with neferine. Analysis of the effect of the change in trans-epidermal water loss (TEWL), erythema, blood flow, hydration, and ear thickness in an atopic-dermatitis-like phenotype in BALB/c mice. Values represent the mean ± SEM from at least six independent experiments. ^#^ *p* < 0.05; ^##^ *p* < 0.01 compared with the no-treatment condition; * *p* < 0.05 and ** *p* < 0.01 compared with the DNCB-induced group.

**Figure 8:**
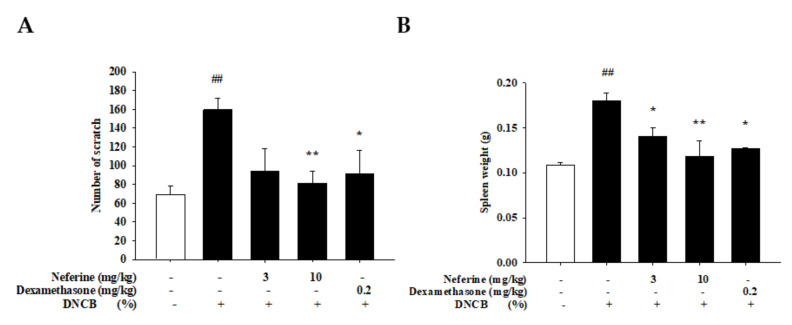
(**A**) Analysis of the effect of the change in the number of scratches in BALB/c mice with an atopic-dermatitis-like phenotype. (**B**) Effect of neferine on the weight of the spleen. Values represent the mean ± SEM from at least six independent experiments. ^##^ *p* < 0.01 compared with the no-treatment condition; * *p* < 0.05 and ** *p* < 0.01 compared with the DNCB-induced group.

AD has a variety of symptoms, including erythema, dryness, edema, and cracked and itchy skin [22]. Therefore, we used a multifunctional skin physiology detection system, a non-contact laser blood flow meter, and a scratching behavior detector to evaluate the effects of neferine and dexamethasone on the physiological parameters of the skin surface of DNCB-induced mice. Before the start of the experiment, a baseline value test was performed, including TEWL, erythema, skin moisture, blood flow, number of scratches, and ear thickness (Figure 7). After inducing the first stage of sensitization to Day 9, the above-mentioned physiological values of the DNCB-induced group had increased significantly. This indicated that the barrier function of the mice had been damaged by external stimulation. In addition, we also determined the condition of the skin stratum corneum. The physiological value of the skin moisture content also tended to decrease day by day, which represents a loss of skin moisture. Our data also show that the thickness of the right ear of the mice in the DNCB experimental group increased significantly. However, the TEWL, erythema, blood flow, ear thickness, and scratching number were reduced in the neferine and dexamethasone treatment groups (Figure 7 and Figure 8A). The effect of 10 mg/kg of neferine on the mice in the DNCB experimental group was better on the 15th day, and the effect of 3 mg/kg of neferine was similar to that found in the dexamethasone 0.2 mg/kg experimental group. These results indicate that a neferine intervention can significantly improve the damage to the mouse skin barrier and reduce the symptoms related to skin and ear inflammation and scratching reactions in mice with atopic dermatitis. The spleen is an important lymphatic organ in the body. It has the functions of producing hematopoiesis, filtering blood, removing aging blood cells, and participating in a variety of immune responses. The spleen is the largest immune organ and can be enlarged when in an inflamed state. We observed that the weight of the spleen increased significantly due to inflammation and swelling in the DNCB experimental group. Compared with the neferine and dexamethasone drug treatment groups, the weight of the spleen had decreased. Our data indicate that neferine has the effect of inhibiting inflammation and swelling of the spleen (Figure 8B).

### 2.7. Neferine Inhibits the Phosphorylation of p38 and ERK Induced by DNCB in BALB/c Mice

**Figure 9:**
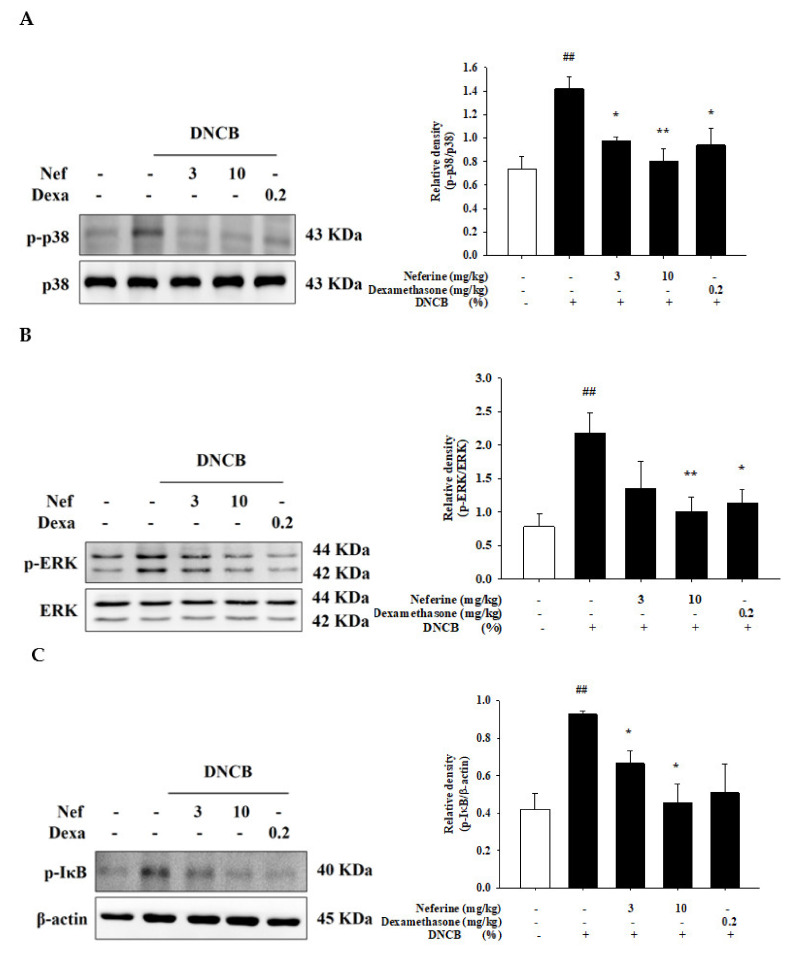
Effect of neferine on p38 (**A**), ERK (**B**), and IκB (**C**) activation in BALB/c mice with an atopic-dermatitis-like phenotype. Western blots were analyzed quantitatively. Values represent the mean ± SEM from at least six independent experiments. ^##^ *p* < 0.01 compared with the no-treatment condition; * *p* < 0.05 and ** *p* < 0.01 compared with the DNCB-induced group.

DNCB activates p38 and ERK and thereby stimulates TNF-α release and phenotypic changes through different signal transduction pathways [23]. Therefore, we explored whether neferine can participate in the regulation of the p38 and ERK pathways. In the experimental group with DNCB-induced AD, we found that the p38 protein was activated and had a significant phosphorylation effect. When the group was treated with neferine and dexamethasone drugs during the experiment, a significant decrease in p38 phosphorylation was observed. Among the treatments, 10 mg/kg of neferine had the best effect (Figure 9A). Similarly, we also found that the DNCB experimental group promoted a significant increase in the phosphorylation of the ERK protein. After treatment with neferine and dexamethasone, the phosphorylation of ERK was significantly reduced (Figure 9B). NF-κB is a transcription factor that can promote the production of cytokines, such as IL-6. Under normal circumstances, NF-κB binds to IκB in the cytoplasm and exists in an inactive form. After activation, it will be phosphorylated and degrade IκB, and NF-κB will enter the nucleus for gene transcription. The NF-κB pathway is also regulated by the MAPK pathway and is considered to be a key mediator in the pathogenesis of atopic dermatitis. Therefore, we evaluated whether mice with DNCB-induced inflammation treated with neferine were affected. The experimental results show that the IκB in the DNCB experimental group was significantly phosphorylated, and that the neferine and dexamethasone drug treatment group significantly reduced IκB phosphorylation (Figure 9C).

### 2.8. Neferine Downregulates the mRNA Expression of Pro-Inflammatory Cytokines and Chemokines Induced by DNCB

**Figure 10:**
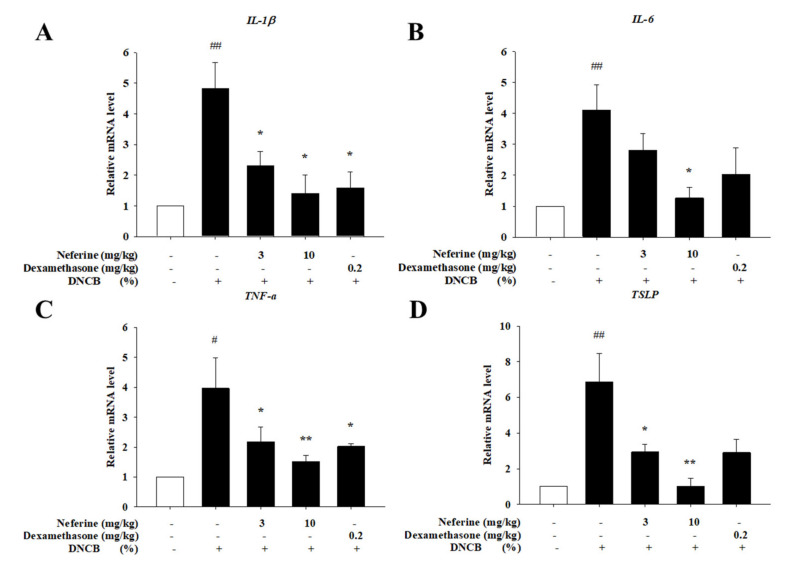
Effect of neferine on cytokine mRNA expression in BALB/c mice with an atopic-dermatitis-like phenotype. Total RNA was isolated and an mRNA analysis was performed via real-time RT-PCR. (**A**) IL-1β, (**B**) IL-6, (**C**) TNF-α, and (**D**) TSLP. Values represent the mean ± SEM from at least six independent experiments. ^#^ *p* < 0.05; ^##^ *p* < 0.01 compared with the no-treatment condition; * *p* < 0.05 and ** *p* < 0.01 compared with the DNCB-induced AD group.

Various pro-inflammatory cytokines and chemokines contribute to inflammation in the pathogenesis of AD [24]. The production of these cytokines and chemokines is accompanied by the infiltration of inflammatory cells (Th2) into the skin and expands the inflammatory response. Therefore, we detected IL-1β, IL-6, TSLP, and TNF-α and explored whether neferine can downregulate the expression of mRNA. In the group treated with neferine and dexamethasone, it was observed that the mRNA expression levels of IL-1β, IL-6, TSLP, and TNF-α were significantly suppressed (Figure 10). These experimental results prove that neferine can effectively downregulate the expression of cytokines to inhibit the expression of cytokines produced by DNCB-induced atopic dermatitis.

## 3. Discussion

Proinflammatory cytokines and chemokines secreted by keratinocytes, such as TNF-α, IFN-γ, IL-1β, IL-6, IL-8, TARC (CCL17), MDC (CCL22), and RANTES, play a very important role in dermatitis [25,26,27]. They may induce skin inflammation and allergic reactions and expand the entire immune response. The inhibition of their production may be an effective therapeutic target for AD. Therefore, our research focused on the cytokines, chemokines, and related signal transduction pathways produced by atopic dermatitis. In vitro results show that neferine has an anti-inflammatory effect on keratinocytes stimulated by TNF-α/IFN-γ. In vivo results show that neferine has anti-inflammatory and anti-allergic effects on BALB/c mice stimulated by DNCB. Therefore, we conclude that neferine has potential application in the treatment of inflammatory skin diseases such as atopic dermatitis.

AD is a heterogeneous disease that may be triggered by individual genetic or environmental factors. Whether it is induced by gene–gene or gene–environmental effects, it is considered to be the underlying pathological mechanism of skin inflammation caused by damage to the skin barrier, abnormal cells, and the accumulation and infiltration of T cells into the dermis [28]. A number of studies have shown that IFN-γ and TNF-α stimulate epidermal keratinocytes, thereby activating many signal transduction pathways and participating in the promotion of inflammation [29,30,31]. Therefore, IFN-γ/TNF-α stimulation is usually used as an induction method for in vitro anti-inflammatory skin experiments. When the epidermal barrier is damaged, keratinocytes will be stimulated, causing many cytokines (IL-1β, IL-33, and TSLP) and chemotactic factors (TARC and MDC) to be expressed [32,33]. These cellular mediators expand and activate the immune response mediated by innate lymphoid cells (ILCs) and type II helper T cells (Th2) in the dermis [34]. TSLP induces dendritic cells to express the OX40 ligand, which can stimulate the production of IL-4, IL-5, and IL-13 after binding to its receptor. Dendritic cells receive antigens released by damaged keratinocytes, causing the infiltration of CD8+ T cells, which produce the second type of immune cytokine. CD8+ T cells that produce the second type of immune cytokine infiltrate and activate B cells and promote the release of immunoglobulin E (IgE) [35,36]. Except for keratinocytes, which release TSLP when inflamed, their immune response to Th2 is due to the initiation of dendritic-cell-specific activities [35]. Previous studies have shown that TSLP produced by skin keratinocytes effectively activates mast cells [37]. Other cytokines, such as IL-6, are considered to be important cellular mediators of host immunity and inflammation. IL-6 can be formed by fibroblasts, monocytes, endothelial cells, and keratinocytes. In a human study, it was found that the T cells of AD patients spontaneously produce large amounts of IL-6. In addition, IL-6 can also increase the expression of IgE receptors (FcεRI) and the production of histamine, which is closely related to allergic reactions. IL-8 is one of the main factors produced in many inflammatory reactions. It directly affects downstream immune cells and can be secreted by different cells. Keratinocytes are a rich source of IL-8, and IL-8 from the epidermis has biological activity [38]. Therefore, IL-8 can activate a variety of intracellular signal transmission pathways. Among them, two cell surface receptors, CXCR1 and CXCR2, are expressed in human neutrophil CD8+ T cells and natural killer cells (NK cells) [39], which induce the chemotaxis of neutrophil granulocytes, mast cells, macrophages, endothelial cells, and keratinocytes [40]. Our results show that TNF-α and IFN-γ stimulate human keratinocytes to induce the expression of large amounts of inflammatory factors (IL-1β, IL-6, IL-8, TSLP, TARC, MDC, and RANTES). The mRNA levels were significantly downregulated after neferine pretreatment. These results indicate that neferine has an immunomodulatory effect on the skin.

The activation of both pathways is involved in the expression of many pro-inflammatory genes [21,41]. Previous studies have shown that p38 in MAPKs is mainly involved in inflammation-related reactions, and its activation is related to the expression of CXCL-8, TARC, and GM-CSF. After external stimulation, the phosphorylated p38 will also affect the transmission of downstream signal pathways. In addition, when bronchial endothelial cells are stimulated by lysophosphatidic acid (LPA), they will increase their secretion of cytokines, such as IL-6 and IL-8, through the phosphorylation of JNK and p38 [42,43]. MAPKs could regulate the downstream NF-κB/IκBα signaling pathway. The level of expression of pro-inflammatory cytokines depends on the activation of the transcription factor NF-κB in keratinocytes. When the IκB protein is phosphorylated and degraded, NF-κB will be released and translocate to the nucleus, where it activates target genes. Many cytokines and chemokines are produced and regulate the immune response and inflammation [43]. Our results show that when TNF-α/IFN-γ is added to human keratinocytes, the MAPK signaling pathway is rapidly activated, and at the same time affects and initiates downstream NF-κB signaling. It was observed that the phosphorylation of these related proteins was inhibited after pretreatment with neferine. These results show that neferine can indeed effectively inhibit the molecular mechanism of inflammation and allergic reactions caused by atopic dermatitis.

Skin barrier function also plays a very important role in atopic dermatitis. Inflammatory cytokines are secreted and produced at the skin injury site, which can cause severe inflammation and itching [44]. Studies have shown that TSLP is the initial stimulus of the skin inflammation and immune response that occur in keratinocytes. TSLP can directly stimulate and regulate itching. TSLP receptors are found in the C fibers of primary sensory afferent neurons of the skin. TSLP secreted by keratinocytes makes the C fibers sensitive, and the body can recognize pathological itching [45,46]. Studies have shown that the increase in TEWL may occur earlier than the clinical manifestation of AD [47,48]. From the results of in vitro experiments, it can be known that neferine acts on keratinocytes to exert anti-inflammatory and anti-allergic effects and affect the immune response. In order to evaluate the effectiveness of neferine in vivo, neferine was also used in mouse AD animal models. The induction of DNCB transformed the first T cell immune response of mice into the second T cell immune response [48,49]. Our research results show that the mice had an obvious itching response, and the tissue morphology and skin appearance exhibited a lot of erythema and epidermal thickening and scaling in the DNCB group. Regarding other physiological parameters of the skin, increased TEWL and decreased hydration were also observed. Further results also confirmed the molecular and biochemical mechanisms, indicating that inflammation-related cytokines, such as IL-1β, IL-6, TNF-α and TSLP, the IκB protein, the MAPK pathway, p38, the ERK protein, and the NF-κB pathway were all phosphorylated. In the neferine group, a dose of 3 mg/kg was found to significantly improve these target molecules, and a dose of 10 mg/kg was found to achieve the best effect. Therefore, we believe that neferine has the potential to be used to effectively treat atopic dermatitis.

## 4. Materials and Methods

### 4.1. Animals

Male BALB/c mice (eight weeks old) were purchased from the National Laboratory Animal Center in Taiwan. The animals were maintained in a standard laboratory under a 12-h light/dark cycle in a temperature (21 ± 2 °C), humidity (50 ± 20%), and filtered laminar air-flow-controlled room in the animal facility at the Animal Center of Fu Jen Catholic University, New Taipei City, Taiwan. All mice were allowed access to water and food ad libitum. The experiment was approved by the Institutional Animal Care and Use Committee of Fu Jen Catholic University (approval number A10703).

### 4.2. MTT Assay

The human immortalized keratinocytes (HaCaT cells) were a gift from J.Y. Fang, Chang Gung University, Taiwan. HaCaT cells belong to a human keratinocyte cell line that has been widely used as an in vitro model of proliferative epidermis [50]. MTT measures the metabolic activity of cells. The cells were seeded in a 24-well culture plate at 5 × 10^4^/well. After 24 h of drug treatment, 300 μL of MTT (3-(4, 5-dimethylthiazol-2-yl)-2, 5-diphenyl-2H-tetrazolium) was added per well. The cells were placed in an incubator at 37 °C for 2–4 h and then dissolved in crystal violet with DMSO. An ELISA reader was used to measure the absorbance intensity at a wavelength of 550 nm as a cell viability test.

### 4.3. Quantitative Polymer Chain Reaction (qPCR)

HaCaT cells were planted in a 3.5 cm culture dish. After the cells reached 90% confluence, the cells were grown in a static state for 24 h. After the cells were pretreated with neferine for 20 min, they were stimulated with TNF-α/IFN-γ for 1 h or 24 h, respectively. The cells were scraped off and centrifuged (16,000× *g*, 10 min, 4 °C), and the supernatant was extracted. RNA was purified using a total RNA isolation kit (GeneDireX^®^, Vegas, NV, USA). According to the operating procedure of the iScript™ cDNA Synthesis Kit (BIO-RAD, Hercules, CA, USA), reagents were added in order and operated upon according to the indicated conditions to convert the RNA into cDNA. Furthermore, PowerUp™ SYBR™ Green Master Mix (Applied Biosystems™, Waltham, MA, USA) was used. A total of 7.5 μL of ddH_2_O, 2 μL of cDNA, 0.25 μL of forward and reverse primer, and 10 μL of SYBR GREEN were added and mixed uniformly. The primer sequences are shown in Table 1. Then, the RNA was quantified using an ABI StepOnePlus™ Real-time PCR System.

### 4.4. Western Blot Assay

Western blotting was used to analyze the changes in the various proteins in cells. HaCaT cells were seeded in a 3.5 cm culture dish. After the cells reached 90% confluence and were starved for 24 h, they were pretreated with neferine for 20 min and then stimulated with TNF-α/IFN-γ for 30 min or 1 h, respectively. After scraping, the cells were pulverized by ultrasound and centrifuged (13,200 rpm, 10 min, 4 °C). After centrifugation, the supernatant was taken and the protein was quantified with a Pierce protein assay kit (Pierce, Rockford, IL, USA). About 20–40 μg of protein was electrophoresed on 10% SDS-polyacrylamide gel and then electroporated with a PVDF membrane. After the transfer was completed, the PVDF membrane was put into TBS-T (Tris-buffered saline/0.05% tween 20) solution containing 5% skimmed milk powder and shaken for 1 h to avoid non-specific binding. Then, the PVDF membrane was washed with TBS-T three times (10 min each time). Then, primary antibodies (in a 1:1000 dilution) were added. The PVDF membrane was left overnight at 4 °C and then washed three times with TBS-T for 10 min each time. After adding secondary antibodies (diluted to 1:1000) for 1 h, the PVDF membrane was washed three times with TBS-T for 10 min each time. Finally, the developer was added, and the membrane was placed in a chemical luminescence extraction system (BIOSTEP Celvin^®^) for shooting.

### 4.5. Dinitrochlorobenzene (2,4-dinitrochlorobenzene, DNCB)-Induced Atopic-Dermatitis-Like Skin Inflammation in Mice

First, the mice were divided into five groups: a control group, a DNCB group (negative group), a neferine (3 mg/kg and 10 mg/kg) with DNCB group, and a dexamathasone (0.2 mg/kg) with DNCB group (positive group). Dexamethasone is a corticosteroid hormone that can reduce swelling and allergic reactions. In this in vivo experiment, both neferine and dexamathasone were dissolved in DMSO, while DNCB was dissolved in 75% ethanol by an ultrasonic shock. The former was given by intraperitoneal injection, and 100 µL and 20 µL of the latter was applied to the skin on the back and the right ear, respectively. Three days before the experiment, the mice were anesthetized, the back hair was removed, and a small measuring magnet (SCT-MAG-TF) was embedded on the back of each mouse’s back feet. After being left to rest for three days, it was confirmed that the mice were in good physical condition and that the skin on the dorsal depilation area was normal. The experiment was then started. Before the experiment, the relevant physiological values of mouse skin parameters were measured, including TEWL, erythema, skin moisture, blood flow, ear thickness, and number of scratches. During the experiment, photographs were taken to record the changes in the appearance of the skin and ears. Because the temperature and humidity in the environment have a great influence on the parameters to be measured on the skin surface, when evaluating the physiological parameters of the skin, the whole process was carried out in a chamber at a constant temperature and humidity. The first stage (Days 1–4) was the period of allergic atopic dermatitis. After measuring the basic physiological values of mice in the DNCB group, the neferine (3 and 10 mg/kg) and DNCB experimental group, and the dexamethasone (0.2 mg/kg) and DNCB experimental group, 1% DNCB was evenly applied to the skin on the back and the right ear. On the fifth day, neferine and dexamathasone were injected intraperitoneally. The second stage (Day 5 to Day 14) was the re-induction of atopic dermatitis. We evenly smeared 0.5% DNCB on the skin on the back and the right ear of the mice in the three experimental groups. The next day, we tested and recorded the skin’s physiological values and took pictures. After the mice were euthanized with carbon dioxide (CO_2_) on the 15th day, the dorsal skin tissue and spleen were removed for subsequent experimental analysis.

### 4.6. Statistical Analysis

Sigma-Plot software (Version 10.0) was used for statistical analysis. All data are expressed as mean ± SEM. Statistical significance was assessed by an unpaired, two-tailed Student’s t test. *p* values less than 0.05 and 0.01 were considered to indicate a significant difference and are denoted by an asterisk (*) and a pound sign (#), respectively.

## 5. Conclusions

According to the results of the in vitro study, cytokines and activators related to the pathogenicity of atopic dermatitis, such as TNF-α and IFN-γ, can induce inflammation and allergic reactions. In human keratinocytes, TNF-α and IFN-γ can induce the secretion of many cytokines (IL-1β, IL-6, IL-8, and TSLP) and chemotactic factors (TARC, MDC, and RANTES), and can increase the phosphorylation of IκB and NF-κB in the MAPK pathway and p38, JNK, and ERK in the NF-κB pathway. Neferine exerts anti-inflammatory effects by inhibiting the expression of chemokines and pro-inflammatory cytokines and the MAPK activation induced by TNF-α/IFN-γ.

In the in vivo study, we found that neferine improved the inflammatory damage, redness, and desquamation of the skin on the back of the mice, significantly reduced the TEWL, erythema, blood flow rate, ear thickness, and itching, and increased the skin moisture content when used to treat DNCB-induced atopic dermatitis. These experimental results indicate that neferine has anti-atopic dermatitis activity and helps to inhibit AD-related inflammation and allergic reactions. In the future, we will further explore the application of neferine in different inflammatory and allergic skin diseases to determine whether it can relieve and inhibit pathological symptoms.

## Figures and Tables

**Table 1:** Primer sequences for RT-qPCR.

Genes	Primers	Sequence (5′-3′)
hIL-1β mIL-1β	Forward	CTC TCA CCT CTC CTA CTC ACT TGG ACC TTC CAG GAT GAG GAC A
hIL-1β mIL-1β	Reverse	ATC AGA ATG TGG GAG CGA AT GTT CAT CTC GGA GCC TGT AGT G
hIL-6 m IL-6	Forward	CGA GCC CAC CGG GAA CGA AA AGT TGC CTT CTT GGG ACT GA
hIL-6 m IL-6	Reverse	GGA CCG AAG GCG CTT GTG GAG TCC ACG ATT TCC CAG AGA AC
IL-8	Forward	ACT GAG AGT GAT TGA GAG TGG AC
	Reverse	AAC CCT CTG CAC CCA GTT TTC
hTNF-α mTNF-α	Forward	ATGGGCTCCCTCTCATCAGT GGT GCC TAT GTC TCA GCC TCT TTT
hTNF-α mTNF-α	Reverse	GAAATGGCAAATCGGCTGAC GCC ATA GAA CTG ATG AGA GGG AG
hTSLP m TSLP	Forward	TATGAGTGGGACCAAAAGTACCG AGC TTG TCT CCT GAA AAT CGA G
hTSLP m TSLP	Reverse	GGGATTGAAGGTTAGGCTCTGG AGG TTT GAT TCA GGC AGA TGT T
MDC	Forward	GTT GTC CTC GTC CTC CTT GC
	Reverse	GGA GTC TGA GGT CCA GTA GAA GTG
TARC	Forward	GTC TTG AAG CCT CCT CAC CC
	Reverse	GGA TCT CCC TCA CTG TGG CT
RANTES	Forward	AGT GTG TGC CAA CCC AGA GA
	Reverse	AGC AAG CAG AAA CAG GCA AA
GAPDH	Forward	CTG CTC CTC CTG TTC GAC AGT
	Reverse	CCG TTG ACT CCG ACC TTC AC

## Data Availability

Not applicable.

## Supplementary Materials

The following are available online at https://www.mdpi.com/article/10.3390/ijms22158237/s1.
