# Supplementary material for: Effect of Neferine on DNCB-Induced Atopic Dermatitis in HaCaT Cells and BALB/c Mice
Source: Int J Mol Sci. 2021 Jul 30;22(15):8237. doi: 10.3390/ijms22158237 (PMC8348662; doi:10.3390/ijms22158237)
Supplement: Supplementary file 1 [file ijms-22-08237-s001.zip › ijms-1320976-supplementary.pdf]

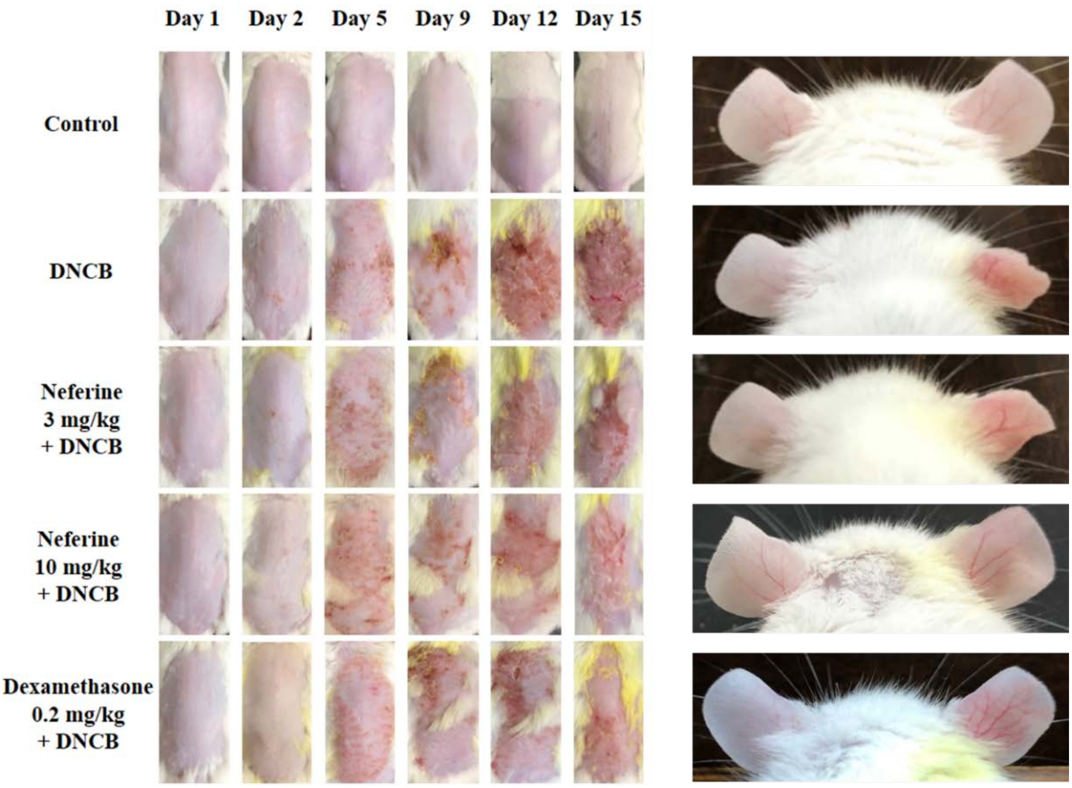

Figure S1. The effect of neferine on the skin and ear appearance in DNCB-induced BALB/c mice. Phenotypic presentation of mouse skin after 15 days treatment.
